# Cyberchondria, Health Anxiety, and Sleep Quality: An Observational Cross-Sectional Study of Adults with and Without Psychosomatic Disorders

**DOI:** 10.3390/healthcare14101356

**Published:** 2026-05-15

**Authors:** Reda Ebrahim Mohamed Elashram, Ali Mohammed Ali Al-Basiouni Bashshar, Ahmed Samir Sedik Abo-Bakr, Ali Marzouq Al-Ghamdi

**Affiliations:** 1 Special Education Department, College of Education, Imam Mohammad Ibn Saud Islamic University (IMSIU), Riyadh 11623, Saudi Arabia; 2 Faculty of Education in Tafahna Al-Ashraf, Al-Azhar University, Mit Ghamr 35822, Egypt; alibachar@azhar.edu.eg; 3 Faculty of Education, Minia University, Minia 61519, Egypt; ahmed.aboubakr@mu.edu.eg; 4 Educational Administration Department, College of Education, Imam Mohammad Ibn Saud Islamic University (IMSIU), Riyadh 11623, Saudi Arabia; aalghamdi@imamu.edu.sa

**Keywords:** cyberchondria, health anxiety, sleep quality, psychosomatic disorders, digital health, Saudi Arabia

## Abstract

**Highlights:**

**What are the main findings?**

- Cyberchondria is highly prevalent (56.78%) among Saudi adults.
- Poor sleep quality affects 56.9% of the studied population.

**What are the implications of the main findings?**

- Psychosomatic disorders significantly amplify cyberchondria and health anxiety.
- Strong positive correlations exist between cyberchondria, anxiety, and sleep quality.

**Abstract:**

**Background/Objectives**: The increasing reliance on the Internet for health information has contributed to the emergence of cyberchondria, a phenomenon closely associated with health anxiety and potentially linked to sleep disturbances. Evidence remains limited in the Saudi context, particularly regarding differences between individuals with and without psychosomatic disorders. **Methods**: A cross-sectional observational study was conducted among 1224 Saudi adults (535 with psychosomatic disorders and 689 without). Data were collected using validated instruments, including the Cyberchondria Severity Scale (CSS-12), Short Health Anxiety Inventory (SHAI-18), and Pittsburgh Sleep Quality Index (PSQI). Statistical analyses included Pearson correlation coefficients and two-way ANOVA. **Results**: The prevalence of cyberchondria was 56.78%, health anxiety 38.76%, and poor sleep quality 56.9%. Significant positive correlations were observed between cyberchondria, health anxiety, and poor sleep quality across both groups, with stronger associations among individuals with psychosomatic disorders. Two-way ANOVA revealed a significant main effect of clinical status on all variables and a significant effect of sex on health anxiety, with higher levels among females. **Conclusions**: Findings highlight a significant interplay between cyberchondria, health anxiety, and sleep quality, particularly among individuals with psychosomatic disorders. These results underscore the need for targeted public health interventions addressing digital health behaviours and mental health.

## 1. Introduction

Over the past decade, health information-seeking behaviours have shifted dramatically, with the Internet emerging as the primary source for the public [1]. This transformation has fostered the rise of cyberchondria—a compulsive, excessive pattern of online health information searching that intensifies psychological distress and health-related fears [2]. Cyberchondria is closely associated with health anxiety, defined as persistent and disproportionate worry about having or developing a serious illness [3,4]. The repercussions of both extend to sleep quality: empirical evidence consistently demonstrates that heightened anxiety and psychological stress contribute directly to sleep disturbances [5,6].

Understanding these interactions is particularly relevant in the Saudi context, where Internet usage is rising [7], and cyberchondria appears increasingly prevalent among adults [8]. Yet comparative research examining cyberchondria, health anxiety, and sleep quality simultaneously within a clinically stratified framework—distinguishing adults with and without psychosomatic disorders—remains entirely absent from the Arabic-language literature and exceptionally scarce in the broader cross-cultural evidence base. This gap is clinically consequential: psychosomatic disorders may amplify the cognitive, behavioural, and physiological pathways linking these variables, yet this possibility has not been empirically tested in the Saudi context. The present study was designed to address this gap directly, contributing the first Arabic-language comparative investigation of these three constructs within a clinically stratified sample.

Accordingly, the present study aims to: (1) estimate the prevalence of cyberchondria, health anxiety, and sleep disturbances among Saudi adults; (2) examine correlational relationships among these variables within each clinical group; and (3) investigate differences based on gender, clinical group, and their interaction. Findings are expected to advance the understanding of psychophysiological mechanisms and inform evidence-based preventive and therapeutic interventions.

### 1.1. Theoretical Framework and Literature Review

#### 1.1.1. Cyberchondria

Cyberchondria represents a multidimensional psychological construct characterised by compulsive online searching for health-related information, accompanied by excessive health anxiety and catastrophic interpretation of obtained information [9]. Although not explicitly classified in the Diagnostic and Statistical Manual of Mental Disorders, Fifth Edition (DSM-5), it is conceptually situated within the spectrum of anxiety disorders [1]. Severity is commonly assessed using the Cyberchondria Severity Scale (CSS), which encompasses compulsion, distress, excessiveness, and reassurance-seeking dimensions [10]. The validated Arabic version (CSS-12) has demonstrated acceptable psychometric properties within the Saudi context [1].

Cyberchondria is strongly associated with health anxiety, functioning as a behavioural mechanism that perpetuates the anxiety cycle: health anxiety motivates information searching, which in turn exacerbates anxiety in a self-reinforcing loop that drives further searching [11,12]. Recent Saudi evidence confirms elevated cyberchondria levels in adult and student populations, particularly on the excessiveness and reassurance-seeking subscales [8,13], and documents its co-occurrence with somatic symptom burden [13]. Higher cyberchondria severity is also significantly associated with impaired sleep quality [6,14].

#### 1.1.2. Health Anxiety

Health anxiety is a multidimensional psychological construct characterised by persistent and excessive preoccupation with physical health and fear of contracting serious illnesses, often based on the misinterpretation of bodily sensations [3,15].

Clinically, this concept has undergone a significant shift in the DSM-5, where the term hypochondriasis was replaced by two more precise classifications: somatic symptom disorder (SSD), emphasising distressing somatic symptoms accompanied by excessive thoughts and feelings, and illness anxiety disorder (IAD), characterised by intense fear of illness in the absence of substantial somatic symptoms [3,16].

These modern classifications are grounded in a deeper understanding of underlying cognitive mechanisms, most notably catastrophic misinterpretation of normal bodily sensations and heightened sensitivity to internal cues [4]. Dysfunctional beliefs act as drivers for repetitive reassurance-seeking behaviours, which, while temporarily reducing stress, ultimately reinforce health-related fears and hinder long-term psychological adaptation [17]. Distinguishing health anxiety from disease phobia is crucial: the former involves a broad and generalised worry about health, whereas the latter is limited to specific, avoidant fear of a particular illness [18].

#### 1.1.3. Sleep Quality

Poor sleep quality—encompassing chronic difficulties initiating or maintaining sleep and non-restorative sleep despite sufficient opportunity—carries significant functional consequences, including chronic fatigue, impaired cognitive processes, and increased emotional reactivity [5,19]. Standardised instruments such as the Pittsburgh Sleep Quality Index (PSQI) are widely used to determine clinical thresholds for poor sleep quality [20,21].

The mechanistic link between anxiety and sleep impairment is well-established: excessive anxiety activates sympathetic nervous system activity, physiologically manifesting as difficulty relaxing—a process that directly explains the close association between health anxiety, cyberchondria, and impaired sleep efficiency [6,22].

#### 1.1.4. The Dynamic Interplay Between Cyberchondria, Health Anxiety, and Sleep Quality

The cognitive–behavioural model developed by Salkovskis and Warwick [15] provides the foundational framework: dysfunctional health beliefs activate catastrophic misinterpretation of ordinary bodily sensations, driving compulsive safety behaviours—including online health searching—as responses intended to reduce tension [4]. Cyberchondria amplifies this cycle: initial searches triggered by perceived illness probability temporarily reduce anxiety, yet the excessive and often contradictory nature of information encountered escalates psychological distress and perpetuates the cycle through metacognitive fusion and difficulties in emotion regulation [11,23,24,25].

This anxiety loop directly impairs sleep quality through a bidirectional and circular set of pathways. Persistent health-related anxiety triggers sustained sympathetic nervous system activation, impairing the mental and physical relaxation necessary for sleep onset [5,22]. Simultaneously, cyberchondria—particularly through intrusive pre-sleep health-related cognitions—delays sleep onset and disrupts sleep maintenance. In the reverse direction, poor sleep quality reduces psychological resilience and intensifies compulsive digital health-seeking behaviours, while deteriorating sleep lowers pain thresholds and increases psychological vulnerability, further exacerbating psychosomatic symptoms [6,14,26]. These relationships are therefore best understood as circular and mutually reinforcing rather than unidirectional.

The inclusion of sleep quality as a focal outcome variable in this tripartite framework is grounded in three convergent mechanistic pathways. First, the hyperarousal model of insomnia (Riemann et al., 2010) [27] posits that sustained cognitive and physiological arousal—the precise condition perpetuated by cyberchondria’s ruminative, repetitive health-seeking cycle—constitutes a primary etiological pathway to sleep-onset difficulties and fragmented sleep maintenance. Second, within the cognitive–behavioural framework of health anxiety (Salkovskis & Warwick, 1986) [15], the intrusive pre-sleep cognitions characteristic of health-anxious individuals—including rehearsal of illness scenarios encountered during online searching and anticipatory symptom worry—directly impede the cognitive deactivation that is neurobiologically necessary for sleep initiation. Third, from a psychosomatic perspective, individuals with clinically relevant somatic symptom burden experience elevated allostatic load and dysregulated hypothalamic–pituitary–adrenal axis reactivity (Huang et al., 2025) [22], physiologically manifesting as hypervigilance and fragmented sleep architecture. These three mechanisms converge to predict that cyberchondria and health anxiety are neurobiologically, as well as psychologically, linked to impaired sleep quality—particularly in individuals with psychosomatic disorders, where all three pathways may operate simultaneously.

These interactions gain further clinical complexity in the context of somatic symptom and related disorders, such as SSD and IAD [3]. Individuals with these disorders exhibit persistent catastrophic symptom interpretation and excessive somatic fixation [18], resulting in sustained sympathetic nervous system activation that physiologically accounts for the strong link between pathological anxiety and chronically impaired sleep efficiency in these populations [22,28].

### 1.2. Research Problem and Hypotheses

#### 1.2.1. Research Problem

The present study addresses the need to understand the complex cognitive–behavioural–physiological mechanisms that influence the mental and physical health of Saudi adults, particularly in light of the increasing reliance on the Internet as a primary source of health information. This challenge is exemplified by cyberchondria—a repetitive and excessive pattern of online health information searching that leads to heightened psychological distress and exacerbated health-related fears [2]. Although individual components of this triad have been examined in Arabic-speaking contexts, no previous study conducted in Saudi Arabia or the broader Arab world has investigated cyberchondria, health anxiety, and sleep quality simultaneously within a clinically stratified comparative framework. This methodological gap is particularly important because psychosomatic disorders may influence all three outcomes concurrently, yet this potential clinical moderating role remains untested in the Saudi population. To address this gap, the present study adopts a cross-sectional observational design. Such designs are appropriate for estimating prevalence patterns [13,14] and allow for comparisons between adults exhibiting illness behaviour [28] and those without such manifestations. Accordingly, this design provides an important empirical foundation for subsequent longitudinal and intervention-based research. Previous literature has reported inconsistent findings regarding the relationships among gender, health anxiety, sleep disturbances, and cyberchondria. Meta-analytic evidence suggests that females tend to report higher levels of health anxiety across different cultural contexts, possibly due to differences in interoceptive sensitivity, hormonal influences on stress reactivity, and socialisation toward health-monitoring behaviours [29]. Epidemiological studies have likewise documented higher rates of insomnia among women [30]. However, findings from Saudi studies examining the relationship between gender and cyberchondria remain inconclusive and inconsistent [7,31]. In addition, the psychosomatic literature indicates that individuals with psychosomatic disorders may experience amplified somatic symptom perception, catastrophic cognitive interpretations, and chronic sympathetic activation [18,22], factors that may contribute to elevated levels of cyberchondria, health anxiety, and sleep disturbances. Nevertheless, this potential clinical influence has not been sufficiently examined within the Saudi context. Therefore, further investigation is needed to clarify these relationships among Saudi adults.

#### 1.2.2. Study Hypotheses

Based on the identified research gaps, the study aims to: (1) estimate the prevalence and severity of cyberchondria, health anxiety, and sleep disturbances; (2) examine correlational relationships among these variables within each clinical group; and (3) investigate differences attributable to gender, clinical group, and their interaction.

The study tests the following hypotheses:

1. Cyberchondria, health anxiety, and sleep quality disturbances will be prevalent at notable levels among study participants.
2. Statistically significant positive correlations will exist between cyberchondria, health anxiety, and impaired sleep quality.
3. Statistically significant differences will exist in mean scores based on gender, clinical group, and their interaction. Clarifying note on the analytical role of the SSS-8: The Somatic Symptom Scale-8 (SSS-8) was employed exclusively as a stratification instrument to operationalise the psychosomatic/non-psychosomatic group distinction (see Section 2.3.4), in conjunction with a self-reported clinically confirmed diagnosis. The SSS-8 was not included as a dependent variable in any analytical model; accordingly, no hypothesis was formulated regarding SSS-8 total scores. Its role is that of a group-classification criterion, not an outcome measure.

## 2. Materials and Methods

### 2.1. Method and Design

The present study employed a descriptive correlational comparative approach, utilising an observational cross-sectional design, which allows for the examination of multiple variables at a single point in time. This design is particularly appropriate for estimating the prevalence of cyberchondria, health anxiety, and impaired sleep quality within a defined population at a specific time point [32]. Although cross-sectional designs are inherently limited by their inability to establish causal relationships or determine temporal precedence among variables, they remain effective for identifying concurrent associations and assessing group differences—such as comparisons between individuals with and without psychosomatic disorders—and thus constitute a suitable and justifiable methodological choice for the objectives of the present study [33].

Data were collected using an anonymous online questionnaire distributed via Internet-based platforms, such as Google Forms, to ensure broad accessibility and participation from individuals across different regions.

### 2.2. Participants

The minimum required sample size was determined a priori using power analysis (G*Power software (Version 3.1.9.7; Heinrich-Heine-Universität Düsseldorf, Düsseldorf, Germany), assuming a small-to-moderate effect size (0.10), a significance level of α = 0.05, and a statistical power of 99%, yielding a minimum required sample of 912 participants. To enhance data quality and compensate for incomplete or invalid responses, the final sample size was increased to 1224 adult participants residing in Saudi Arabia (*M*_age = 26.22 years, *SD* = 6.15).

**Inclusion Criteria**
  1. Age 18 years or older;
  2. Residency in Saudi Arabia;
  3. Ability to read Arabic;
  4. Provision of informed consent.
**Exclusion Criteria**
  1. Incomplete questionnaire responses;
  2. Illogical response patterns (e.g., selecting the same response option for all items).

The final sample consisted of 713 females (58.3%) and 511 males (41.7%). For comparative purposes, participants were classified into two groups based on: (1) a clinically confirmed diagnosis provided by a qualified professional, and (2) their scores on the Somatic Symptom Scale-8 (SSS-8). Participants scoring ≥ 8 on the SSS-8 were classified as having psychosomatic disorders, in accordance with established diagnostic criteria [34].

Accordingly, 535 participants (43.7%) were classified as having psychosomatic disorders, while 689 participants (56.3%) were classified as non-psychosomatic. Age distribution indicated that 41.4% of participants were between 18 and 22 years, 59.4% were unmarried, and 50.3% had attained a university-level education (see Table 1).

Participants were recruited via non-probability convenience sampling, disseminated across multiple social media platforms (Twitter/X, WhatsApp community groups, and institutional announcement channels) to maximise geographic distribution across Saudi regions. While this approach yielded a large, geographically diverse sample within a compressed data-collection window, it entails inherent self-selection bias: individuals with higher digital literacy and greater Internet engagement are systematically more likely to encounter and complete online surveys, potentially over-representing those with greater cyberchondria tendencies relative to the broader Saudi adult population. Additionally, a precise response rate could not be calculated because of the anonymous broadcast distribution method used. These constraints are considered in the interpretation of prevalence estimates presented in Section 3.1 and are addressed further in the Limitations section (Section 4.2).

### 2.3. Measures

The study relied on four standardised self-report measures, all of which have been previously validated and psychometrically supported within the Arabic/Saudi context, ensuring cultural appropriateness and measurement accuracy.

#### 2.3.1. Cyberchondria Severity Scale (CSS-12)

The study relied on the short form of the Cyberchondria Severity Scale (CSS-12). This is a self-report measure consisting of 12 items, designed to assess the severity of the phenomenon across four core dimensions: compulsion, distress, excessiveness, and reassurance-seeking [1,35,36]. Responses are recorded on a five-point Likert scale [1,2,3,4,5], with total scores ranging from 12 to 60; higher scores reflect greater levels of cyberchondria. The scale’s suitability for the local context was ensured by using a validated Arabic version, which employed back-translation to ensure linguistic and cultural accuracy [1,7].

Previous Saudi studies have demonstrated high reliability for this version, with Cronbach’s alpha coefficients ranging between 0.88 and 0.882 [1,7]. Internal consistency analyses revealed adequate item-total correlations (0.586 to 0.807, all *p* < 0.01) and strong item-subscale correlations (0.778 to 0.890), with the four subscales demonstrating robust associations with the total scale: Compulsion (r = 0.876), Excessiveness, (r = 0.814), Distress (r = 0.852). and Reassurance-Seeking (r = 0.873).

In the current study, confirmatory factor analysis (CFA; AMOS (Version 26.0; IBM Corp., Armonk, NY, USA); validation subsample, *n* = 296) supported the hypothesised four-factor structure (CFI = 0.97, TLI = 0.957, GFI = 0.944, NFI = 0.951, IFI = 0.971, RMSEA = 0.05; standardised factor loadings = 0.62–0.92, all *p* < 0.01). Internal consistency was excellent (α = 0.920 for the total scale; subscale α = 0.766–0.892). Full CFA model matrices, item-level correlations, and split-half reliability coefficients are reported in Supplementary Table S1.

#### 2.3.2. Short Health Anxiety Inventory—SHAI

Health anxiety was assessed using the Short Health Anxiety Inventory (SHAI-18). The scale consists of 18 items, with responses rated on a 4-point Likert scale (0 to 3), designed to measure two main dimensions: Illness Likelihood and Negative Consequences [15,37]. The Arabic version (A-SHAI) has demonstrated validity and reliability in a Saudi sample, with internal consistency reliability of the full scale reaching α = 0.85 [3].

In the current study, CFA (AMOS v.26; *n* = 296) confirmed the hypothesised two-factor structure (CFI = 0.915, TLI = 0.90, GFI = 0.925, NFI = 0.903, IFI = 0.917, RMSEA = 0.047; standardised factor loadings = 0.352–0.661, all *p* < 0.01). Internal consistency was good (total α = 0.822; Illness Likelihood α = 0.806; Negative Consequences α = 0.795). Full psychometric statistics are provided in Supplementary Table S1.

#### 2.3.3. Pittsburgh Sleep Quality Index (PSQI)

Sleep quality was assessed using the Pittsburgh Sleep Quality Index (PSQI), a globally recognised self-report measure consisting of 19 items evaluating sleep quality over the past month [30]. The instrument yields scores for seven components (Subjective Sleep Quality, Sleep Latency, Sleep Duration, Habitual Sleep Efficiency, Sleep Disturbances, Use of Sleeping Medication, and Daytime Dysfunction), combined into a global score ranging from 0 to 21, with a score of 5 or above indicating poor sleep quality [38].

Previous studies have demonstrated acceptable reliability for the scale, with Cronbach’s alpha values ranging between 0.73 and 0.914 [20,30]. The scale also showed excellent reliability in the current study (Cronbach’s alpha = 0.877). Furthermore, the unidimensional factor structure of the PSQI has been validated in prior research [30].

In the current study, CFA (AMOS v.26; *n* = 296) supported the seven-component structure (CFI = 0.985, TLI = 0.972, GFI = 0.988, NFI = 0.933, IFI = 0.986, RMSEA = 0.029; standardised factor loadings = 0.273–0.827, all *p* < 0.01). Internal consistency was excellent (α = 0.877). Full psychometric statistics are provided in Supplementary Table S1.

#### 2.3.4. Somatic Symptom Scale (SSS-8)

The Somatic Symptom Scale-8 (SSS-8) is a reliable standardised instrument for assessing somatic symptom burden, characterised by strong psychometric properties that make it effective in differentiating individuals with psychosomatic disorders from those without [13,34]. Its use aligns with the diagnostic framework for somatic symptom disorder as outlined in the DSM-5 [39]. The scale consists of eight items rated on a Likert scale from 0 (not at all) to 4 (very much), yielding total scores ranging from 0 to 32.

The validity of the SSS-8 has been established across multiple languages, including German, Chinese, Greek, Russian, Korean, Japanese, and Arabic, demonstrating its international applicability [40,41,42,43,44]. Alalawi et al. [45] utilised the Arabic version to assess somatic symptom disorder within primary healthcare settings. The scale consists of eight items rated on a Likert scale from 0 (not at all) to 4 (very much), yielding total scores ranging from 0 to 32, with scores ≥ 8 indicating a clinically relevant symptom burden [34,45].

The SSS-8 has demonstrated excellent internal consistency, with Cronbach’s alpha coefficients ranging from 0.80 to 0.85 internationally [44,46], and 0.82 in the Arabic context [13]. It has also shown high test–retest reliability [42]. Regarding construct validity, the scale exhibits strong correlations with measures of anxiety and depression, comparable to longer instruments such as the Patient Health Questionnaire–15 (PHQ-15) [47], supporting its suitability as an efficient screening tool for both clinical and research settings [22,28].

The SSS-8 ≥ 8 threshold for classifying participants into the psychosomatic group is grounded in the empirical validation work of Gierk et al. (2014) [34], who established this cutoff as corresponding to a “medium” somatic symptom burden—a level associated with clinically meaningful functional impairment and elevated healthcare utilisation in large representative community samples. It is critical to emphasise that the SSS-8 serves here as a validated continuous-severity screening and stratification instrument, not as a standalone diagnostic tool. Group classification in the current study required the convergence of two criteria: (a) a clinically confirmed diagnosis provided by a qualified healthcare professional, as self-reported by the participant; and (b) an SSS-8 score ≥ 8. This dual-criterion approach enhances the validity of group assignment beyond any single-measure classification scheme while acknowledging that neither criterion alone constitutes a gold-standard diagnostic procedure.

Note on the analytical role of the SSS-8: This scale was employed exclusively as a stratification instrument to operationalise the psychosomatic/non-psychosomatic group distinction; it was not included as a dependent variable in any correlational or group-comparison model. Accordingly, no hypothesis was formulated regarding SSS-8 total scores.

In the current study, CFA (AMOS v.26; *n* = 296) supported the one-factor structure (CFI = 0.977, TLI = 0.977, GFI = 0.975, NFI = 0.95, IFI = 0.93, RMSEA = 0.05; standardised factor loadings = 0.52–0.682, all *p* < 0.01). Internal consistency was good (α = 0.827). Full psychometric statistics are provided in Supplementary Table S1.

### 2.4. Statistical Analysis

Data were statistically analysed using IBM SPSS Statistics (Version 29.0; IBM Corp., Armonk, NY, USA). The analyses comprised: (1) descriptive statistics (means and standard deviations) to summarise the data; (2) Pearson’s product–moment correlation coefficient (r) to examine bivariate relationships among the study variables; and (3) a two-way analysis of variance (two-way ANOVA) to test group differences (clinical vs. non-clinical) and sex, as well as their interaction effects, with effect sizes reported using partial eta squared (η^2^p). Statistical significance was set at α = 0.05.

The analytical plan was pre-specified and registered with the Institutional Review Board before data collection. All analyses reported herein correspond directly to the three pre-specified study hypotheses. No post hoc or exploratory analyses beyond those hypotheses are reported, in keeping with the descriptive–correlational–comparative nature of the study design.

### 2.5. Ethical Considerations

This study was conducted in accordance with the ethical principles outlined in the Declaration of Helsinki. The study protocol received ethical approval from the Institutional Review Board (IRB) of the first author’s affiliated university prior to data collection (Approval No. 26870/1447, dated 22 October 2025). All participants were provided with comprehensive information regarding the study objectives and procedures and were informed of their right to withdraw at any time without penalty. Electronic informed consent was obtained before participation. To ensure confidentiality, data were collected anonymously, and participation was entirely voluntary. The authors declare no conflicts of interest. All procedures complied with relevant institutional and national ethical standards.

## 3. Results

This section presents the findings of the statistical analyses conducted to test the study hypotheses.

### 3.1. Prevalence Rates and Mean Scores of Cyberchondria, Health Anxiety, and Sleep Quality by Clinical Group

The overall sample exhibited moderate levels of cyberchondria, with 56.78% of participants falling within the moderate-to-high range (*M* = 34.07, *SD* = 8.68), and moderate levels of health anxiety, with a prevalence rate of 38.76% (*M* = 20.93, *SD* = 9.08). Regarding sleep quality, 56.9% of participants (*n* = 696) scored at or above the PSQI clinical cutoff (≥5), indicating a substantial prevalence of sleep disturbances within the study sample. These findings provide empirical support for the first hypothesis of the study.

**Figure 1:**
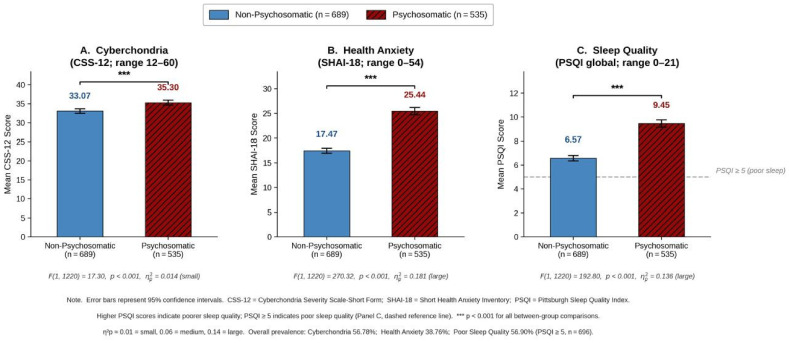
Mean Scores on Cyberchondria (CSS-12), Health Anxiety (SHAI-18), and Sleep Quality (PSQI) by Clinical Group (Non-Psychosomatic, *n* = 689 vs. Psychosomatic, *n* = 535). Note. Error bars represent 95% confidence intervals. CSS-12 = Cyberchondria Severity Scale–Short Form (range 12–60); SHAI-18 = Short Health Anxiety Inventory (range 0–54); PSQI = Pittsburgh Sleep Quality Index global score (range 0–21; higher scores indicate poorer sleep quality). The dashed reference line in Panel (**C**) indicates the PSQI ≥ 5 clinical cutoff for poor sleep quality. Blue bars = non-psychosomatic group; red bars = psychosomatic group. Panels (**A**–**C**) show statistically significant between-group differences (all *p* < 0.001). Effect sizes: Cyberchondria η^2^p = 0.014 (small); Health Anxiety η^2^p = 0.181 (large); Sleep Quality η^2^p = 0.136 (large). *** *p* < 0.001.

Figure 1 presents the mean scores on all three variables disaggregated by clinical group (psychosomatic, *n* = 535 vs. non-psychosomatic, *n* = 689), with 95% confidence intervals. Participants with psychosomatic disorders obtained consistently higher mean scores across all three variables compared to the non-psychosomatic group: cyberchondria (*M* = 35.30 vs. *M* = 33.07), health anxiety (*M* = 25.44 vs. *M* = 17.47), and sleep quality (*M* = 9.45 vs. *M* = 6.57; higher PSQI scores indicate poorer sleep quality). This groupwise visual presentation communicates the differential burden of each variable across clinical groups—a pattern that directly addresses Hypothesis 1—and supplements the overall prevalence figures with information about the magnitude and direction of between-group differences.

### 3.2. Correlational Relationships Between Cyberchondria, Health Anxiety, and Poor Sleep Quality

The most clinically salient finding of the correlation analysis was the consistently stronger associations between cyberchondria and poor sleep quality among individuals with psychosomatic disorders (total-scale r = 0.33, 95% CI [0.24, 0.41]) compared to the non-psychosomatic group (r = 0.27, 95% CI [0.19, 0.34]), suggesting that psychosomatic status amplifies the concurrent co-occurrence of compulsive health-seeking behaviour and sleep disturbance—although the direction of this relationship cannot be established from cross-sectional data (see Discussion, Section 4).

Pearson correlation analyses were conducted to examine the associations between cyberchondria dimensions, health anxiety, and sleep quality separately for adults with and without psychosomatic disorders (see Table 2). Correlation strength was interpreted following Cohen’s guidelines.

The results revealed statistically significant positive associations (*p* < 0.001) between all dimensions of cyberchondria and both health anxiety and sleep quality across the two groups. Notably, the magnitude of the correlation coefficients was higher among individuals with psychosomatic disorders compared to their non-affected counterparts. Specifically, the correlation between total cyberchondria and health anxiety was stronger in the psychosomatic group (r = 0.46) than in the non-psychosomatic group (r = 0.42). A significant positive association was also observed between cyberchondria and poor sleep quality (i.e., higher PSQI scores), indicating that increased compulsive health-related online searching was associated with poorer sleep quality. Collectively, these findings provide empirical support for the second hypothesis of the study.

### 3.3. Sex and Group Differences (Psychosomatic vs. Non-Psychosomatic) in Cyberchondria Dimensions, Health Anxiety Dimensions, and Sleep Quality: A Two-Way ANOVA

To examine the statistical significance of differences according to sex (male vs. female) and group (individuals with psychosomatic disorders vs. those without) in cyberchondria, health anxiety, and sleep quality, a two-way analysis of variance (two-way ANOVA) was conducted. The assumptions underlying the analysis were evaluated before model estimation. Given the large sample size (*n* = 1224), the data were considered sufficiently robust to potential minor violations of the normality assumption. Descriptive statistics by gender and psychoso-matic status are reported in Table 3.

As shown in Table 4, a significant main effect of group (psychosomatic vs. non-psychosomatic) was observed across all study outcomes. Participants with psychosomatic disorders reported higher levels of cyberchondria, F(1, 1220) = 17.30, *p* < 0.001, η^2^p = 0.014, higher health anxiety, F(1, 1220) = 270.32, *p* < 0.001, η^2^p = 0.181, and poorer sleep quality, F(1, 1220) = 192.80, *p* < 0.001, η^2^p = 0.136, compared to non-psychosomatic participants.

Effect sizes warrant explicit clinical interpretation. The large effect of clinical group on health anxiety (η^2^p = 0.181) indicates that psychosomatic status accounts for approximately 18% of the variance in health anxiety scores—a magnitude classified as large by conventional benchmarks (η^2^p ≈ 0.14), and one carrying clear clinical significance: psychosomatic disorder is not merely a statistically distinguishable correlate of health anxiety but a substantive determinant of its severity. Similarly, the large effect on sleep quality (η^2^p = 0.136) positions clinical status as a primary driver of sleep disturbance in this sample. In contrast, the small effect of clinical group on cyberchondria (η^2^p = 0.014) indicates that while group differences in online health-seeking are reliable and significant, they are shaped by a broader constellation of individual, contextual, and dispositional factors that extend beyond clinical status alone—a distinction that warrants systematic investigation in future research.

A significant main effect of sex emerged for health anxiety only, F(1, 1220) = 19.44, *p* < 0.001, η^2^p = 0.016 (small effect), with females reporting higher levels than males. No significant main effects of sex were found for cyberchondria or sleep quality. All sex × group interactions were non-significant (ps > 0.05), indicating that group-related differences were consistent across males and females.

Overall, these findings underscore the salient role of psychosomatic status in shaping health-related cognitive, emotional, and behavioural outcomes, and provide empirical support for the third hypothesis of the study.

## 4. Discussion

The present study examined the prevalence of cyberchondria, health anxiety, and sleep disturbances among Saudi adults, their interrelationships, and differences according to sex and psychosomatic status, including interaction effects. Overall, substantial prevalence was observed for cyberchondria (56.78%) and sleep disturbances (56.86%), alongside significant positive associations among cyberchondria, health anxiety, and poor sleep quality, with stronger correlations in the psychosomatic group.

An important interpretive caveat applies to all findings reported here: the cross-sectional design of the present study constrains inference to concurrent association; temporal precedence and causal direction cannot be established. The reverse causal pathway—whereby poor sleep quality, by depleting psychological resilience and heightening affective reactivity, precipitates increased reliance on compulsive online health-seeking as a maladaptive coping strategy—is not only theoretically plausible but is independently supported by longitudinal evidence [14,26]. Accordingly, the associations reported here reflect concurrent co-occurrence within a dynamic feedback system rather than unidirectional predictive relationships. Longitudinal and experimental designs are required to establish directionality and causality with confidence, and all interpretations below are framed accordingly.

Moderate cyberchondria levels indicate widespread engagement in online health-related information seeking, consistent with prior Saudi studies (52.6%; [13]) and evidence of predominantly moderate-to-high levels among Saudi participants [7]. Similarly, Abdulrahman et al. [8] reported elevated cyberchondria among Saudi university students, particularly in excessive searching and reassurance-seeking, with higher scores in married and divorced individuals. Higher prevalence has also been observed regionally, e.g., among Egyptian medical students (85.8%; [16]), highlighting the influence of intensive Internet and smartphone use. These rates exceed those in some international contexts, such as India, where severe cyberchondria was reported in only 4.4% of participants [48], indicating cultural and contextual factors in its manifestation.

Despite moderate cyberchondria and relatively low health anxiety (38.76%), over half of the participants reported poor sleep quality, aligning with global evidence among young adults and university populations (18.5–46.38%; [6,14,49]). These findings underscore sleep quality as a sensitive outcome, susceptible to cognitive and emotional strain even without clinically elevated health anxiety.

Correlation analyses supported the second hypothesis, revealing significant positive associations between cyberchondria, health anxiety, and sleep disturbances. This aligns with the cognitive–behavioural model of health anxiety, which conceptualises cyberchondria as a maladaptive coping mechanism that perpetuates anxiety cycles [11,15]. Compulsive online searching to obtain reassurance often intensifies distress, creating a self-reinforcing cycle [2,4]. Bidirectional influences were observed between cyberchondria and sleep quality [14], as intrusive health-related thoughts impair relaxation, particularly pre-sleep [5]. In individuals with psychosomatic disorders, elevated allostatic load may exacerbate sleep fragility, while poor sleep amplifies cognitive vulnerability and compulsive health-related searching [6]. These processes are consistent with evidence linking cyberchondria and health anxiety to heightened physiological arousal via sympathetic activation, impeding sleep initiation [22].

Two-way ANOVA revealed a robust main effect of psychosomatic status across cyberchondria, health anxiety, and sleep quality, with individuals diagnosed with psychosomatic disorders reporting significantly higher cyberchondria and health anxiety, as well as poorer sleep quality. These findings support the third hypothesis and align with psychosomatic frameworks emphasising catastrophic interpretation of bodily sensations and heightened somatic focus [18]. Notably, correlations were stronger in the psychosomatic group, indicating that these disorders may amplify negative interplay among the three outcomes, highlighting the need for targeted interventions.

Regarding sex differences, females reported higher health anxiety, whereas no sex differences were found in cyberchondria or sleep quality. This is consistent with prior evidence of greater bodily sensitivity and catastrophic interpretations among females [1,29] but contrasts with findings of higher cyberchondria in Saudi females [31]. The absence of sex differences in cyberchondria may reflect the normalisation of online health searching as a universal digital behaviour [8]. While females may exhibit greater affective vulnerability, behavioural manifestations of online searching and functional consequences for sleep appear comparable across sexes [7]. No significant sex × group interactions were observed, indicating psychosomatic vulnerability as a primary risk factor irrespective of sex.

Several alternative explanations for the observed prevalence and association patterns warrant consideration. First, the elevated cyberchondria prevalence (56.78%) may partly reflect a post-pandemic legacy effect: the COVID-19 period established and normalised heightened health vigilance and routine online health-information seeking behaviours that may persist as habitual routines even in the absence of an acute pandemic threat. Future studies should include pandemic-related health history as a covariate to disentangle this possibility. Second, the gender difference in health anxiety, while statistically reliable, may partly reflect differential social desirability in symptom endorsement rather than true differences in underlying prevalence, particularly in conservative social environments where males may systematically underreport health-related worries. Studies employing implicit or behavioural indicators of health anxiety would help resolve this interpretive ambiguity.

From a cultural perspective, Saudi Arabia’s healthcare landscape—characterised by limited primary care appointment accessibility, cultural norms that constrain direct mental health consultation-seeking, and among the highest per-capita smartphone penetration rates globally [7]—may create conditions in which online health information seeking functions as a culturally accessible surrogate for professional consultation. This contextual layer may partially account for the higher cyberchondria prevalence in the current sample relative to Western counterparts and represents a dimension that future cross-cultural research should systematically examine.

### 4.1. Theoretical and Practical Implications

This study offers several theoretical contributions. First, the findings support the cognitive–behavioural–somatic framework, linking maladaptive health beliefs, compulsive digital behaviours, and physiological outcomes such as sleep quality. The results further indicate that the relationships among these psychological variables are not strictly linear but are moderated by clinical status, highlighting the need for more sophisticated theoretical models. Additionally, the study provides evidence that cyberchondria is a cross-cultural phenomenon, although its prevalence and associated patterns may vary according to local context.

From a practical perspective, the findings—while constrained to concurrent associations by the cross-sectional design—suggest specific targets for integrated psychosocial intervention. Public health initiatives should promote digital hygiene and safe online health information-seeking behaviours, particularly among high-risk populations, including individuals with psychosomatic disorders. Cognitive–behavioural therapy (CBT) protocols could be adapted to target cyberchondria by incorporating cognitive restructuring of maladaptive health beliefs; strategies to reduce compulsive online searching; digital hygiene techniques, including scheduled Internet-use protocols; and components aimed at improving sleep quality through anxiety management. Importantly, the co-occurrence of large psychosomatic group effects on both health anxiety and sleep quality suggests that clinical programmes serving patients with somatic symptom burden represent a priority setting for integrated digital-health and sleep-quality intervention. The sex difference in health anxiety, while of modest practical magnitude, suggests that intervention content addressing health-related catastrophic cognitions may benefit from sex-sensitive tailoring, particularly for female patients with psychosomatic disorders. These proposals remain to be confirmed through randomised controlled trial designs before implementation.

Furthermore, the results inform policymakers on the integration of digital health programs within primary healthcare services, especially for patients with psychosomatic disorders, to enhance the prevention, early detection, and management of cyberchondria and related health anxiety.

### 4.2. Limitations and Recommendations for Future Research

This study is subject to several methodological limitations that should be considered when interpreting the findings. First, the cross-sectional design limits the ability to infer causal relationships among the variables; although the results support significant associations, the direction of causality remains uncertain. Second, the exclusive reliance on self-report measures may expose the data to social desirability and recall biases. Third, the use of convenience sampling via social media may introduce selection bias and restrict the generalisability of the findings to the broader Saudi adult population, potentially overrepresenting individuals with high Internet usage. Finally, the study did not examine potential mediating variables, such as health literacy and daily Internet usage duration, which may influence the observed relationships.

In light of these limitations, future research should pursue the following specific methodological directions: (1) employ ecological momentary assessment (EMA) designs to capture within-person daily fluctuations in health-seeking behaviour, health anxiety, and sleep quality, enabling micro-temporal causal analysis not possible with cross-sectional data; (2) incorporate objective sleep measurement—including actigraphy or wrist-worn wearable devices—alongside PSQI self-report to improve outcome validity and reduce reliance on subjective recall; (3) evaluate Arabic-adapted CBT protocols specifically targeting cyberchondria within Saudi primary care settings, using randomised controlled trial designs to establish causal efficacy; (4) conduct cross-national comparative studies across Arab populations (Egypt, UAE, Jordan, Morocco) to isolate culturally specific versus pan-Arab predictors and contextual moderators; and (5) include digital health literacy, daily Internet use duration, and smartphone dependence as covariates and potential moderators, given their theoretical relevance to the cyberchondria–health anxiety relationship [7,14].

A particularly important avenue for future research concerns the mechanistic pathways linking these three variables. The present cross-sectional design, with all variables measured simultaneously, cannot determine temporal precedence and therefore cannot support mediation analysis. Testing the hypothesis that health anxiety mediates the relationship between cyberchondria and sleep quality—the mechanistic pathway of cyberchondria → health anxiety → sleep quality—represents a high-priority research question that longitudinal or diary-based designs are uniquely positioned to address. Moderated mediation models examining the role of clinical group membership and gender as boundary conditions of this pathway are equally warranted and would substantially advance the theoretical understanding of these co-occurring phenomena.

Experimental studies are also warranted to evaluate the efficacy of cognitive–behavioural interventions in reducing cyberchondria and health anxiety and their subsequent effects on sleep quality. Cross-cultural comparative studies across different Arab contexts and studies involving clinically diagnosed samples by qualified professionals are also encouraged to gain a deeper understanding of the phenomenon.

## 5. Conclusions

This study provides observational evidence of the substantial concurrent prevalence and significant mutual associations among cyberchondria (56.78%), sleep disturbances (56.86%), and health anxiety (38.76%) in a large Saudi adult sample. These findings are consistent with a model of co-occurring cognitive, behavioural, and functional difficulties, with associations consistently stronger among individuals with psychosomatic disorders—though the direction and causal mechanisms of these relationships cannot be established from the present cross-sectional design and require longitudinal and experimental confirmation.

Importantly, the findings highlight the pivotal role of psychosomatic disorders in amplifying the co-occurrence of these variables, with affected individuals exhibiting markedly higher levels across all studied outcomes and stronger interrelations among them.

These results address a critical knowledge gap in the Arabic and Saudi literature and underscore that cyberchondria should not be viewed as an isolated phenomenon, but rather as part of a complex interplay among cognitive, behavioural, and functional factors.

The findings are consistent with the potential clinical utility of multilevel integrated psychosocial interventions addressing: (1) digital health literacy and responsible online health information-seeking; (2) compulsive online health searching through behavioural and cognitive restructuring strategies; (3) health anxiety via established CBT protocols; and (4) sleep quality through sleep-hygiene and relaxation-based components, particularly among individuals with psychosomatic disorders. These proposals require confirmation through randomised controlled trial designs before they can be considered evidence-based recommendations for routine clinical practice.

Moreover, the study lays the groundwork for future research investigating the causal and mediating mechanisms linking cyberchondria and health outcomes, with an emphasis on the development and evaluation of evidence-based interventions within the Saudi and broader Arab context. The findings also highlight the importance of adopting an integrated psycho–somatic–digital perspective to understand contemporary health challenges associated with digital transformation.

## Figures and Tables

**Table 1:** Demographic characteristics of the participants.

Participant Characteristics		Frequency	Percentage
Gender	Male	511	41.7%
	Female	713	58.3%
Psychosomatic disorders	Without	689	56.3%
	With	535	43.7%
Age	18–22	507	41.42%
	23–27	206	16.83%
	28–32	303	24.75%
	33–37	126	10.29%
	38–42	82	6.7%
Marital status	Not married	727	59.4%
	Married	497	40.6%
Education	High school	488	39.87%
	Undergraduate	615	50.26%
	Postgraduate	121	9.88%

**Table 2:** Pearson correlations between cyberchondria dimensions, health anxiety, and sleep quality by group.

Adults Without Psychosomatic Disorders (*n* = 689)						
Cyberchondria Dimension	Health Anxiety r	95% CI	*p*	Sleep Quality r	95% CI	*p*
Compulsion	**0.22** **	[0.14, 0.30]	<0.001	**0.15** **	[0.08, 0.23]	<0.001
Excessiveness	**0.28** **	[0.21, 0.35]	<0.001	**0.20** **	[0.13, 0.27]	<0.001
Distress	**0.42** **	[0.36, 0.50]	<0.001	**0.25** **	[0.18, 0.32]	<0.001
Reassurance seeking	**0.30** **	[0.24, 0.38]	<0.001	**0.19** **	[0.11, 0.28]	<0.001
Total Cyberchondria	**0.42** **	[0.36, 0.49]	<0.001	**0.27** **	[0.19, 0.34]	<0.001
Adults with Psychosomatic Disorders (*n* = 535)						
Compulsion	**0.42** **	[0.33, 0.49]	<0.001	**0.28** **	[0.20, 0.37]	<0.001
Excessiveness	**0.34** **	[0.26, 0.42]	<0.001	**0.22** **	[0.14, 0.30]	<0.001
Distress	**0.40** **	[0.33, 0.47]	<0.001	**0.29** **	[0.23, 0.38]	<0.001
Reassurance seeking	**0.30** **	[0.23, 0.38]	<0.001	**0.23** **	[0.15, 0.31]	<0.001
Total Cyberchondria	**0.46** **	[0.34, 0.54]	<0.001	**0.33** **	[0.24, 0.41]	<0.001

Note. r = Pearson correlation coefficient. CI = confidence interval. All correlations are two-tailed. Higher sleep quality scores indicate poorer sleep quality. Bolded correlations indicate statistical significance after Bonferroni correction (α = 0.004). ** *p* < 0.01.

**Table 3:** Descriptive statistics for study variables by gender and psychosomatic status.

Variable	Gender	Without Psychosomatic (*n* = 689)		With Psychosomatic (*n* = 535)		Total (*n* = 1224)	
		*M*	*SD*	*M*	*SD*	*M*	*SD*
Cyberchondria	Male	32.96	8.33	34.87	9.45	33.55	8.73
	Female	33.15	8.14	35.6	8.9	34.45	8.63
Health Anxiety	Male	16.08	7.41	24.33	9.32	18.62	8.89
	Female	18.46	6.7	26.24	8.95	22.59	8.86
Sleep Quality	Male	6.46	3.06	9.12	3.72	7.28	3.49
	Female	6.64	3.14	9.69	3.7	8.26	3.77

Note. Higher scores on the PSQI indicate poorer sleep quality. *M* = mean; *SD* = standard deviation.

**Table 4:** Results of two-way ANOVA examining effects of group and gender on study variables.

Variable	Source	Sum of Squares	Mean Square	*F*	*p*	η^2^p
Cyberchondria	gender	56.316	56.316	0.76	0.38	0.001
	Groups	1282.532	1282.532	17.30	<0.001	0.014
	gender × Groups	20.213	20.213	0.27	0.60	<0.001
Health Anxiety	gender	1244.856	1244.856	19.44	<0.001	0.016
	Groups	17,313.679	17,313.679	270.32	<0.001	0.181
	gender × Groups	14.665	14.665	0.23	0.63	<0.001
Sleep Quality	gender	37.949	37.949	3.32	0.069	0.003
	Groups	2203.356	2203.356	192.80	<0.001	0.136
	gender × Groups	9.813	9.813	0.86	0.354	0.001

Note. *n* = 1224. Group refers to psychosomatic status (with psychosomatic symptoms, *n* = 535; without psychosomatic symptoms, *n* = 689). η^2^p = partial eta squared.

## Data Availability

The datasets presented in this article are not readily available due to ethical and privacy restrictions. The data contains sensitive psychological and health-related information. In accordance with the ethical approval granted by the Institutional Review Board (IRB) and the informed consent provided by the participants, public sharing of the raw data is restricted to protect participant confidentiality. Requests to access the datasets should be directed to the corresponding author.

## Supplementary Materials

The following supporting information can be downloaded at: https://www.mdpi.com/article/10.3390/healthcare14101356/s1, Table S1: Full psychometric properties of all instruments (CSS-12, SHAI-18, PSQI, SSS-8)—validation subsample (*n* = 296), including confirmatory factor analysis (CFA) fit indices, standardised factor loadings, item-total correlations, subscale-total correlations, and split-half reliability coefficients.
