# Supplementary material for: Cyberchondria, Health Anxiety, and Sleep Quality: An Observational Cross-Sectional Study of Adults with and Without Psychosomatic Disorders
Source: Healthcare (Basel). 2026 May 15;14(10):1356. doi: 10.3390/healthcare14101356 (PMC13205929; doi:10.3390/healthcare14101356)
Supplement: Supplementary file 1 [file healthcare-14-01356-s001.zip › healthcare-4278173-supplementary.pdf]

Supplementary Table S1

Full Psychometric Properties of All Instruments — Validation Subsample (N = 296)

CFA conducted using AMOS v.26. All factor loadings p < .01 (two-tailed).

Scale / Subscale	*k*	*α*	split-half	Item–Total *r* range	Item–Subscale *r* range	Subscale–Total *r*	Factor Loadings	CFI	TLI	GFI	AGFI	NFI	RFI	IFI	RMSEA	Sig.
**CSS-12 — Cyberchondria Severity Scale (Short Form, 12 items) | 4-Factor Structure | CFA (N = 296)**																
**Total Scale**	**12**	**.920**	**.841**	**.586–.807**	**.778–.890**	**—**	**.62–.92**	**.970**	**.957**	**.944**	**.903**	**.951**	**.928**	**.971**	**.050**	***
**Subscales**																
Compulsion	**3**	**.892**	**.882**	—	—	.876	**.84-.92**	—	—	—	—	—	—	—	—	—
Excessiveness	**3**	**.766**	**.738**	—	—	.814	**.62-.81**	—	—	—	—	—	—	—	—	—
Distress	**3**	**.733**	**.803**	—	—	.852	**.62-.80**	—	—	—	—	—	—	—	—	—
Reassurance Seeking	**3**	**.815**	**.764**	—	—	.873	**.75-.90**	—	—	—	—	—	—	—	—	—
**SHAI-18 — Short Health Anxiety Inventory (18 items) | 2-Factor Structure | CFA (N = 296)**																
**Total Scale**	**18**	**.822**	**.795**	**.316–.779**	**.395–.750**	**—**	**.352–.661**	**.915**	**.900**	**.925**	**.901**	**.903**	**.903**	**.917**	**.047**	***
**Subscales**																
Illness Likelihood	14	**.806**	.764	—	—	.969	**.352-.578**	—	—	—	—	—	—	—	—	—
Negative Consequences	4	**.795**	.711	—	—	.663	**.424-.66**	—	—	—	—	—	—	—	—	—
**PSQI — Pittsburgh Sleep Quality Index (19 items) | 7-Component Structure | CFA (N = 296)**																
**Global Score**	**19**	**.877**	**.832**	**.475–.635**	**—**	**—**	**.273–.827**	**.985**	**.972**	**.988**	**.968**	**.933**	**.906**	**.986**	**.029**	***
**Seven Components (all standardised loadings p < .01)**																
**SSS-8 — Somatic Symptom Scale–8 (8 items) | 1-Factor Structure | CFA N = 296 | STRATIFICATION INSTRUMENT ONLY — not a dependent variable**																
**Total Scale**	**8**	**.827**	**.801**	**.600–.715**	**—**	**—**	**.520–.682**	**.977**	**.977**	**.975**	**.949**	**.950**	**.922**	**.930**	**.050**	***

Recommended fit thresholds: CFI, TLI, GFI, AGFI, NFI, RFI, IFI ≥ .90 (acceptable); ≥ .95 (good). RMSEA ≤ .08 (acceptable); ≤ .05 (good).

Note. N = 296 validation subsample. CFA = confirmatory factor analysis; AMOS v.26. k = number of items. α = Cronbach's internal consistency coefficient. split-half = Spearman–Brown split-half reliability coefficient. Item–total and item–subscale correlations are Pearson r; all significant at p < .01 (two-tailed). Subscale–total r = Pearson correlation between subscale total and overall scale total. Factor loadings = range of standardised CFA factor loadings; all significant at p < .01. CFI = Comparative Fit Index; TLI = Tucker–Lewis Index; GFI = Goodness-of-Fit Index; AGFI = Adjusted GFI; NFI = Normed Fit Index; RFI = Relative Fit Index; IFI = Incremental Fit Index; RMSEA = Root Mean Square Error of Approximation. * Loading range for PSQI applies across all seven components. — = not applicable or computed at scale level only. The SSS-8 was employed exclusively as a group-stratification instrument; it is not a dependent variable in any analytical model. ***** p < .001 for all scales.**
